# Impact of tobramycin on the performance of microbial fuel cell

**DOI:** 10.1186/s12934-014-0091-6

**Published:** 2014-07-04

**Authors:** Wenguo Wu, Keaton Larson Lesnik, Shoutao Xu, Luguang Wang, Hong Liu

**Affiliations:** 1 College of Chemical Engineering, Huaqiao University, Xiamen 361021, China; 2 Department of Biological and Ecological Engineering, Oregon State University, Corvallis, OR, USA

## Abstract

**Background:**

The release of antibiotics into aquatic environments has made the treatment of wastewater containing antibiotics a world-wide public health problem. The ability of microbial fuel cells (MFCs) to harvest electricity from organic waste and renewable biomass is attracting increased interest in wastewater treatment. In this paper we investigated the bioelectrochemical response of an electroactive mixed-culture biofilm in MFC to different tobramycin concentrations.

**Results:**

The electroactive biofilms showed a high degree of robustness against tobramycin at the level of μg/L. The current generation responses of the biofilms were affected by the presence of tobramycin. The inhibition ratio of the MFC increased exponentially with the tobramycin concentrations in the range of 0.1-1.9 g/L. The bacterial communities of the biofilms vary with the concentrations of tobramycin, the equilibrium of which is critical for the stability of electroactive biofilms based-MFC.

**Conclusions:**

Experimental results demonstrate that the electroactive biofilm-based MFC is robust against antibiotics at the level of μg/L, but sensitive to changes in antibiotic concentration at the level of g/L. These results could provide significant information about the effects of antibiotics on the performance MFC as a waste-treatment technology.

## Background

Antibiotics, one of the important group of pharmaceuticals in human and veterinary medicine, are widely used in the prevention and treatment of diseases and have been detected in various aquatic environments, for example, wastewater, surface water, ground water and drinking water [[1]–[3]]. Therefore, the release of antibiotics to the aquatic environment as well as its related environmental issues and public health problems have attracted great attention. Biological treatment, the use of bacteria and other microorganisms to remove contaminants by assimilating or oxidizing them, is still regarded as the most common and economical approach for the treatment of contaminants in wastewater [[4]]. Traditionally aerobic treatment consumes large amounts of electrical energy for aeration [[5]]. Anaerobic treatment is generally only suitable for high-strength wastewater streams typically produced by industry [[6]].

Microbial fuel cell (MFC) as a device capable of harvesting electricity from organic waste and renewable biomass, has attracted great interest for wastewater treatment [[5]]. There are various reports about MFCs for biodegradable organics as substrates, for example, glucose, lactate, sucrose, domestic wastewater, brewery wastewater, whey wastewater and starch processing wastewater [[7]–[11]]. Recently MFC technology used for removing toxic and recalcitrant contaminants as substitute substrates in wastewater with much higher chemical oxygen demand (COD) removal efficiencies has drawn great attention [[4],[12]]. The treatment capacity of MFC technology was mainly dependent on the performance of the MFC. In contrast to planktonic cells, wastewater-derived electroactive biofilms show less susceptibility to toxins making MFCs promising for application in the wastewater treatment field [[13]]. Furthermore, the goal of wastewater treatment is COD removal. However, the question that is still largely unaddressed is the effect of the toxins on the performance of electroactive biofilms-based MFC accomplishing removal of COD in pharmaceutical wastewater treatment.

Tobramycin, an aminoglycoside produced by the bacterium *Streptomyces tenebrarius*, is commonly used because of its enhanced effectiveness against infections with the opportunistic pathogen *Pseudomonas aeruginosa* [[14]]. Tobramycin targets the decoding aminoacyl site on the 16S ribosomal RNA, induces miscoding during translation and cell death ensues [[15],[16]]. Herein, the impact of tobramycin on the performance of electroactive biofilms-based MFC was studied. The mixed-culture microorganism community was harvested from wastewater and formed electrochemically active biofilms on the anode generating steady current. An adaptation or change of the microbial community will influence the biofilm structure and the stability of electroactive biofilms-based MFC [[17]]. Hence, the microbial community of the anodic biofilm was analyzed using pyrosequencing and changes of the microbial diversity with different concentrations of tobramycin were determined by denaturing gradient gel electrophoresis (DGGE).

## Results and discussion

### Effects of tobramycin on the performance of MFCs

**Figure 1:**
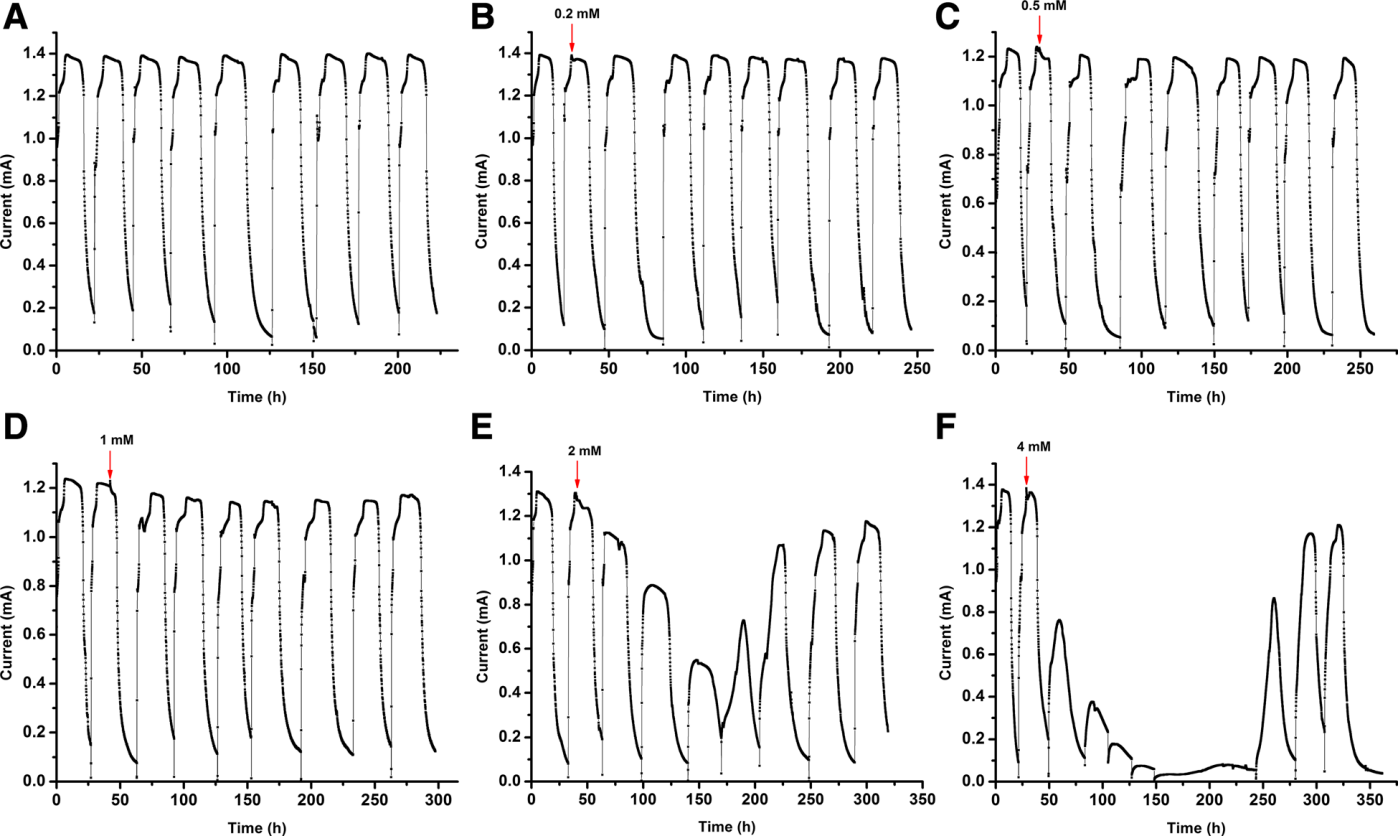
**Current response of electroactive biofilms-based MFC in the absence (A) and presence of tobramycin at 0.2 mM (B), 0.5 mM (C), 1 mM (D), 2 mM (E) and 4 mM (F).** Arrows indicate the addition of tobramycin.

Electrochemically active biofilms were enriched on the anode of MFC, whereupon a repeatable and steady output current in the range of 1.2-1.4 mA was developed at the resistance of 300 Ω without antibiotic (batch 1 in Figure 1). Tobramycin was added into the MFC when the current was stabilized in batch 2. There was also no obvious immediate current decrease after addition of tobramycin at different concentrations (batch 2). However, subsequent batches exhibited distinct current profiles which were more notable at increased tobramycin concentrations. At concentrations of 0.2, 0.5, and 1 mM, inhibition was only exhibited at the beginning of the batch, after which a stable maximum current was maintained. Obvious inhibitions were observed in the MFC with tobramycin concentration of 2 mM at batch 3 and the MFC showed significant inhibition as the concentration increased to 4 mM. However, for the MFCs with tobramycin concentrations of 2 and 4 mM, current recovered 4 and 6 batches post antibiotic addition. These results suggested that some microbial species in the biofilm directly or indirectly facilitating current generation in the biofilm could be recovered from those community members who were not killed by the given antibiotic dose and exposure time, reflected by the recovering stable current for a few batches. The current recovery of MFCs indicated the resistance of the microbial biofilms against the presence of tobramycin in the range of 0.2-4 mM, corresponding to a concentration range of about 0.1-1.9 g/L. Furthermore, pharmaceuticals have been found in surface waters and wastewaters at levels of up to a few μg/L [[18]]. It suggested that there is no response of the biofilm at all in wastewater treatment containing antibiotics in this range. A similar result was also reported that no effect was observed in biofilms-based MFCs in the presence of toxins which were at concentrations an order of magnitude higher than average concentrations in wastewaters [[13]].

### Inhibition ratio of MFCs correlated to tobramycin concentrations

**Figure 2:**
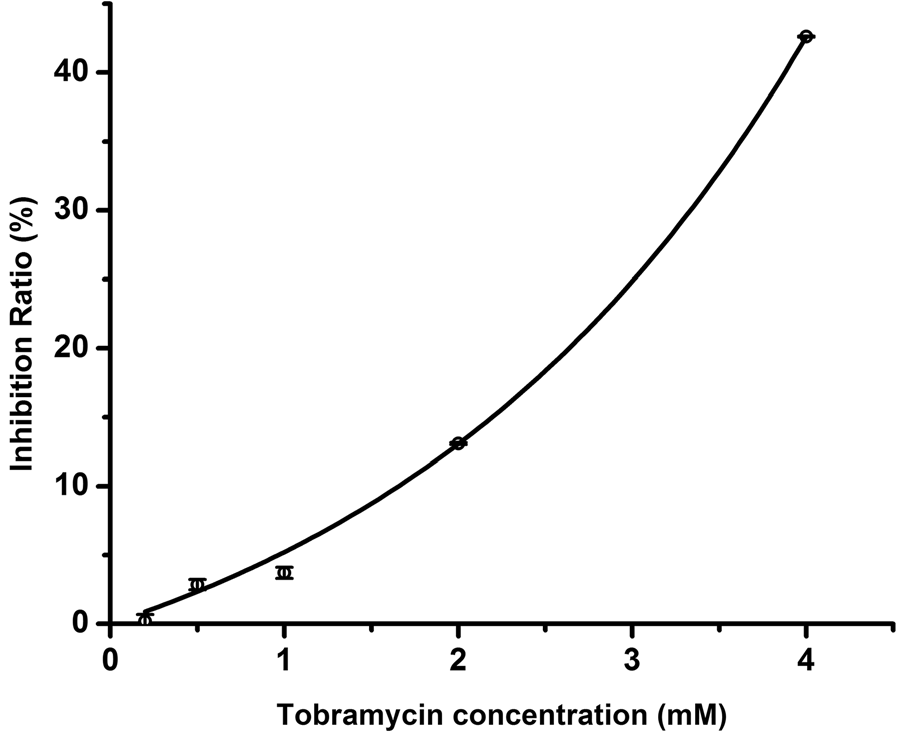
**Plot of the inhibition ratio for the electroactive biofilms-based MFC in response to tobramycin at different concentrations.** Error bars represent relative standard deviations (n = 3).

In order to further explore the electroactive biofilms against antibiotics at levels of g/L, we compared the inhibition ratios of electroactive biofilms-based MFCs in the presence of different concentrations of tobramycin. As shown in Figure 2, tobramycin showed an exponential relationship of the inhibition ratio with the antibiotic concentrations. The regression equation is:

(1) $$y\;=\;-10.3\left(1-e^{0.4x}\right),\;R^{2}=0.99998$$

This indicated that the reaction taking place on the anode of the MFC would be a combination of biofilm kinetics and electrochemical kinetics. The changing of kinetic inhibition of microorganisms in the biofilm would be one reason of the non-linear correlation between tobramycin and inhibition ratio [[19]]. The inhibition ratio of tobramycin drastically increased from 0.2% to 42.6% as the antibiotic concentrations were increased from 0.2 mM to 4 mM. The tobramycin concentration of 4 mM (1870 mg/L) is three orders of magnitude higher than the reported minimal biofilm eliminating concentration (MBEC) of *E. coli* (2 mg/L) measured by the traditional colony-forming unit (CFU) counting method [[20]]. The inhibition of the biofilm at the tobramycin concentration of 4 mM may lead to significantly reduced substrate oxidation rates and subsequent decreased voltage outputs and substrate consumption rates. Experimental results indicated that the electroactive biofilm-based MFC was robust against antibiotics at the level of μg/L but sensitive to changes in antibiotic concentration at the level of g/L. Kim et al. also reported similar results and suggested a novel biomonitoring system using MFCs for the detection of several toxins at the level of mg/L [[21]].

### MFCs exposure to continuously increasing concentration of tobramycin

**Figure 3:**
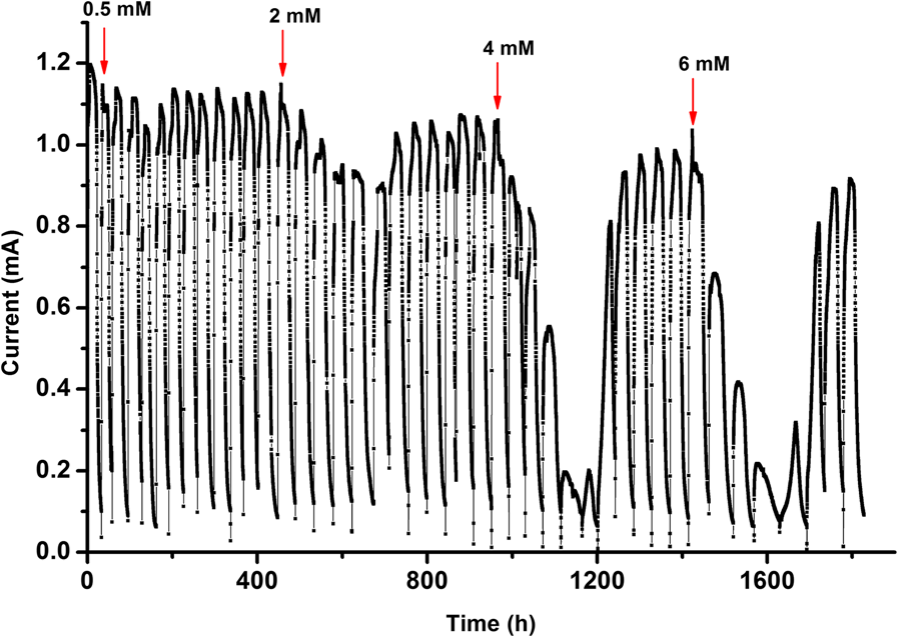
**Current response of electroactive biofilms-based MFC in the presence of continuously increasing concentrations of tobramycin.** Arrows indicate the addition of tobramycin.

To test the continuous effect of tobramycin on MFCs, the performance of the electroactive biofilms-based MFCs in the presence of increasing concentrations of tobramycin was observed (Figure 3). Similar with the result of the MFCs in Figure 1, the current decreased in the batch immediately after the injection of tobramycin and showed greater reduction with the increase of concentration. However, after the addition of tobramycin at the concentration of 6 mM, the significantly inhibited current still recovered to a stable current. It was suggested that some biofilm community members died and some survived during each batch with tobramycin, and then the community regrew in the subsequent batches with no tobarmycin. Moreover, the balance between competition and commensalism in the microbial community of the biofilms in the presence of tobramycin could be one of the reasons for the recovery of a stable current. These results clearly showed the effects of an intermittent shock load and the ability of the anode community to recover from a shock load in the presence of tobramycin. It provides significant information about antibiotics effects on MFC as a waste-treatment technology.

### Effect of tobramycin on the microbial community

**Figure 4:**
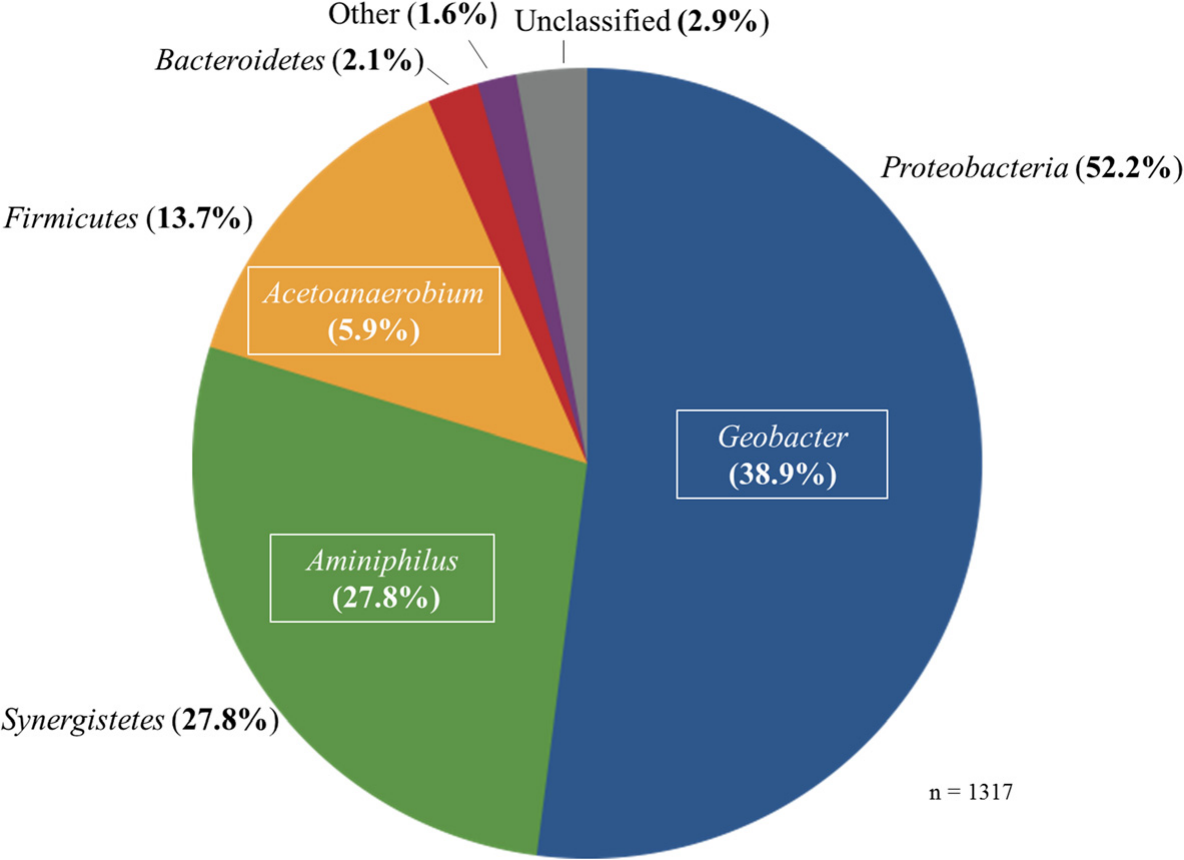
Core microbiome of electroactive biofilms-based MFC with no antibiotic addition (percent of total 16S rRNA reads).

**Figure 5:**
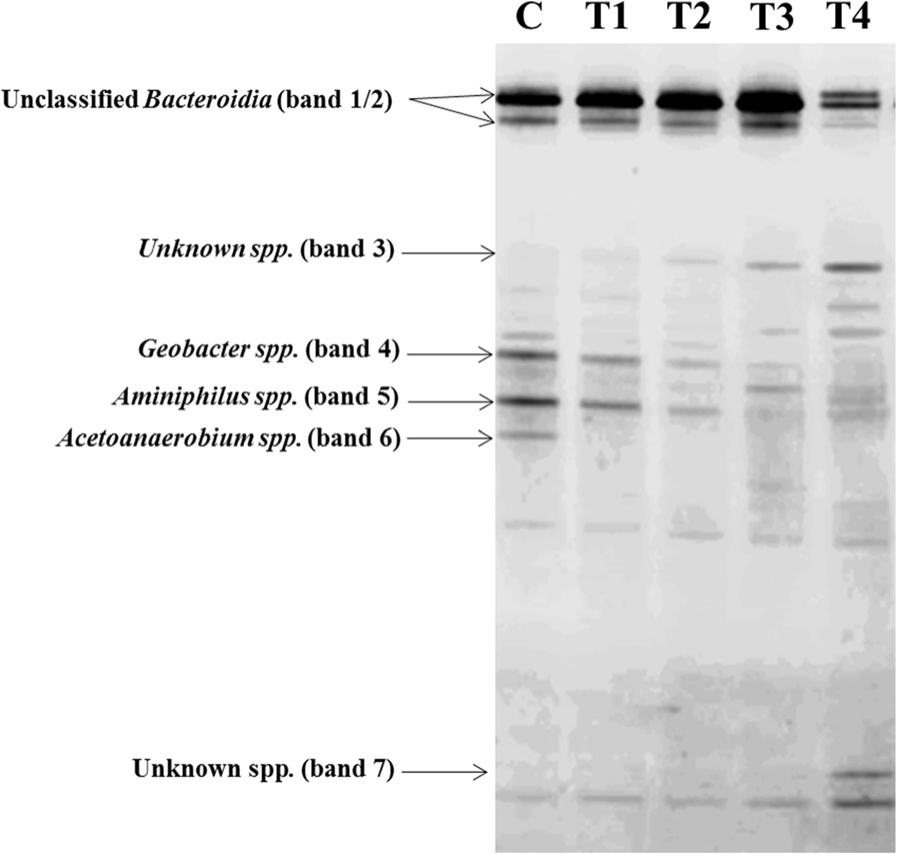
PCR-DGGE analysis of 16S rDNA extracted from the electroactive biofilms-based MFC without tobramycin (lane C) and in the presence of tobramycin at concentrations of 0.5 mM (lane T1), 1 mM (lane T2), 2 mM (lane T3) and 4 mM (lane T4).

The effects of tobramycin concentration on mature acetate-fed MFC anode biofilm communities were analysed using a combination of pyrosequencing and DGGE. Pyrosequencing of the control community (before the addition of tobramycin) yielded 1317 high-quality 16 s rRNA gene reads and following taxonomic assignment revealed the community to be primarily composed of 3 phyla, *Proteobacteria* (52.2%), *Synergistetes* (27.8%), and *Firmicutes* (13.7%) (Figure 4). *Geobacter* spp. made up 38.9% of the community while *Aminiphilus* spp represented 27.8% of 16 s rRNA reads. These populations correspond to band 4 and band 5, respectively (Figure 5). This community structure is consistent with previous analyses of a community maintained in the same laboratory [[22]]. *Geobacter* spp. are well-known for their capability of colonizing and actively respiring anodes resulting in significant current generation [[23]–[25]], while *Aminiphilus* spp. likely play a role as the primary fermentative bacteria, recycling peptones and amino acids into acetate and hydrogen [[26]]. Fermenation-produced acetate could then be ultilized by anode-respiring species such as *Geobacter* spp. The third most common genera, *Acetoanaerobium*, fits the role of a homo-acetogenic hydrogen scavenger [[27]], and along with *Geobacter* and *Aminiphilus* is expected to be involved in a proposed syntrophic interactions that leads to high power densities in MFCs [[28]]. Members of the *Bacteroidia* class were also prominent in on the DGGE gel (Figure 5) (bands 1/2), yet only represented 2.1% of the 16 s pyrosequencing reads with no clear picture of the role they in the present community.

Results following antibiotic introduction show that the microbial communities varied with the increase of tobramycin concentration (Figure 5). As shown in Figure 5, band 4, band 5, and band 6 disappeared at a tobramycin concentration of 4 mM, as tobramycin, being most effective against gram-negative bacteria, was able to reduce populations of *Geobacter* spp., *Aminiphilus* spp., and *Acetoanaerobium* spp. The presence of these members within the community was associated with high power outputs and following their loss at increased antibiotic concentrations concurrent decreases in power outputs were observed (Figure 1). The communities disturbed with high concentrations of tobramycin were the most diverse with unknown bands emerging (Figure 5) (band 3 and 7). Though some similarities can be seen between communities exposed to 3 mM and 4 mM tobramycin, such as the emergence of band 3, other bands were only prominent in one of the treatments (band 7). This suggests that though tobramycin-resistant bacteria were enriched at higher concentrations, it is likely varying community structures will emerge following antibiotic-disruption in contrast to the stability of the community under acetate-fed conditions.

## Conclusions

An electroactive biofilm based-MFC was developed and the electrochemical response of different concentrations of tobramycin on the electroactive mixed-culture biofilms was studied along with the effect to community structure. The electroactive biofilms showed a high degree of robustness against tobramycin at the level of μg/L. The current generated by the electrochemically active biofilm decreased as the tobramycin concentration arrived in the range of 0.1-1.9 g/L and the inhibition ratio increased with the increase of tobramycin concentration. The bacterial communities of the biofilms varied with the concentrations of tobramycin, the equilibrium of which is critical to the stability of electroactive biofilm based-MFCs. These results could provide significant information about the effects of antibiotics on the performance MFC as a waste-treatment technology. In the future, studies on various other microorganisms and antibiotics like sulfadiazine, enoxacin and bacitracin will provide more conclusive results for the treatment of wastewater from pharmaceutical industries.

## Methods

### Construction of electroactive biofilms-based MFCs

A single-chamber MFC was constructed as described previously [[29]]. Briefly, the anode and cathode were placed in parallel on the opposite sides of the chamber (13 mL) with a distance of 1.7 cm. Non-wet proofed carbon cloth (type A, E-TEK, Somerset, NJ, USA; 2 cm^2^) were used as the anode without any treatment. Wet-proofed (30%) carbon cloth (type B, ETEK, Somerset, NJ, USA; 7 cm^2^) was coated with carbon/poly (tetrafluoroethylene) (PTFE) layers on the air-facing side and platinum (0.5 mg/cm^2^ cathode area) with Nafion as binder on the water-facing side, and used as the cathode.

### Operation of electroactive biofilms-based MFCs

Medium used for enrichment and operation of the microbial fuel cell was prepared as previously described [[30]]. Sodium acetate (60 mM) was used as the carbon resource. The medium (8 ml) in the MFC was inoculated with 5 mL of electrochemically active mixed-culture microorganisms, a mixture of gram-positive and gram-negative microorganisms reported in our previous study [[30]]. The electroactive biofilms-based MFC was monitored by a data acquisition system (2700, Keithly, Cleveland, OH, USA), the acetate medium solution was refreshed after each batch until a stable power output was obtained at an external resistance of 300 Ω. When the standard deviation of maximal voltage in the each batch after three batches was within ±5%, 2.6-78 μl of the tobramycin stock solution (1000 mM) was added to the MFC medium to obtain final concentrations in the range of 0.2-6 mM for a single batch, subsequent voltages were then recorded. The following post-antibiotic batches were replaced with the fresh acetate medium solution without tobramycin. Each concentration was run in triplicate.

### Microbial community analysis

Biofilms were separated from the anodes of MFCs treated with different antibiotic concentrations for 20 days (around 10 batches). Bacterial genomic DNA was extracted from the biofilm samples using the DNeasy tissue Kits (Qiagen, CA, USA) according to the manufacturer’s instructions.

The control community with no antibiotic addition was then prepared for pyrosequencing. Primers developed to target the hyper-variable V4 region of the 16S rRNA gene (Cole et al. [[31]]). The 454 adapter sequence (5′- 3′) CCTATCCCCTGTGTGCCTTGGCAGTC the forward primer AYTGGGYDTAAA GNG (Escherichia coli position 563–577). The reverse primers were composed of the adapter sequence followed by the reverse primer sequence, CCGTCAATTCMTTTRAGT (*E. coli* 907–924). Twenty-five microliter PCR reaction volumes were used for optimization followed by 50 μl amplification reactions. A high-fidelity Taq polymerase (Invitrogen Platinum) was used with along with MgSO_4_ (2.5 mM), vendor supplied buffer, BSA (0.1 mg/ml), dNTPs (250 μM), and primers (1 μM). An initial 3-min step at 95°C was followed by 27 cycles of 95°C (45 s), 57°C (45 s), and 72°C (1 min) with a final 3 min extension at 72°C. PCR products were agarose gel purified (2% metaphor in TAE) and bands were extracted with a QIAquick Gel Extraction Kit (Qiagen, Valencia, CA). Gel extracted material was further purified with a Qiagen PCR Cleanup kit and AMPure XP magnetic beads. Quantification of purified PCR product was performed using a Qubit fluorom- eter (Invitrogen, Carlsbad, CA) and qPCR (ABI PRISM 7500 FAST Detection System). Following quantification, libraries were pooled into equimolar amounts. Emulsion PCR and sequencing was performed on a 454 GS Junior pyrosequencer (Roche, Nutley, NJ, USA) at the Center for Genome Research and Biocomputing (CGRB), Oregon State University using titanium reagents and procedures consistent with protocols for unidirectional amplicon sequencing.

Initial quality filtering was performed using MOTHUR, alignment was performed using MUSCLE and subsequent taxonomic identification done using RDP Classifier at an 80% confidence level [[31]–[33]]. Classifier results were then used for community analysis.

All samples were also subject to analysis through DGGE. After nesting 16S ribosomal DNA (rDNA) by using a pair of universal primers: 27 F (5′ -AGAGTTTGATCMTGGCTCAG-3′) and 1492R (5′ -GGTTACCTTTGTTACGACTT-3′) [[34]]. The universal primer set 357 F-GC (5′-GC-clamp-CCTACGGGAGGCAGCAG-3′) and 518R (5′- ATTACCGCGGCTGCTGG-3′) (Invitrogen, Carlsbad, CA, USA) was used to amplify the V3 region of bacteria 16 s rDNA from the extracted genomic DNA. PCR amplification and cycling were performed in a thermocycler (Thermo hybaid, MBS 0.2G, Thermo, MA, USA). DGGE of the PCR products was carried out in a DcodeTM Universal Mutation Detection System (Bio-rad Laboratories, Hercules, CA, USA). Prominent DGGE bands were then excised from the gel and their products amplified using the same PCR system. Amplified products from these bands were then submitted to CGRB for sanger sequencing. Pyrosequencing and DGGE results could then be correlated, allowing for a more comprehensive community analysis.

### Data deposition

Genomic datasets were deposited in the NCBI sequence read archive under accession number. The genomic project can also be accessed in NCBI under Genome Project ID PRJNA252648. (accession, http://www.ncbi.nlm.nih.gov/bioproject?term=PRJNA252648).

### Calculation

The inhibition ratio (I) was calculated as I (%) = 100 × (A_M1_ –A_M2_)/ A_M1_, where A_M1_ was the maximal current in the batch before tobramycin addition, A_M2_ was the maximal current in the following batch after addition of tobramycin.

## Competing interests

The authors declare that they have no competing interests.

## Authors’ contributions

WW carried out the operation of electroactive biofilms-based MFC, participated in the microbial community analysis and drafted the manuscript. KLL carried out the microbial community analysis. SX participated in the construction of MFC and helped to revise the manuscript. LW participated in microbial community analysis. HL participated in its design and coordination and helped to draft the manuscript. All authors read and approved the final manuscript.
